# Validation of the Contextual Sensation-Seeking Questionnaire for skiing and snowboarding among Chinese adult skiers and its relationship with risk-taking behavior

**DOI:** 10.3389/fpsyg.2025.1410930

**Published:** 2025-02-24

**Authors:** Zeyou Guo, Shenmao Gao, Yuanguo Liu, Renfang Zhang, Guangbo Dou

**Affiliations:** ^1^College of Exercise and Health, Shenyang Sport University, Shenyang, China; ^2^Department of Ice and Snow Teaching and Research, Shenyang Sport University, Shenyang, China

**Keywords:** high-risk sport, reliability, skiing injuries, Chinese skiers, risk-taking behavior

## Abstract

**Objective:**

To validate the reliability and validity of the Contextual Sensation-seeking Questionnaire for Skiing and Snowboarding (CSSQ-S) among Chinese adult skiers, and to explore the relationship between sensation seeking and risk-taking behavior.

**Methods:**

Snowball sampling was employed to gather data from 515 individuals, aged between 18 and 40 years old, with at least one year of experience in skiing or snowboarding. Exploratory factor analysis (EFA), confirmatory factor analysis (CFA), and other statistical methods were utilized for data analysis.

**Results:**

The CSSQ-S demonstrated strong factor validity, internal consistency, and construct validity. Sensation seeking was significantly positively correlated with injury frequency, and risk perception mediated this relationship.

**Conclusion:**

The CSSQ-S can be regarded as a reliable and valid tool for measuring sensation-seeking levels and potential injury risks among Chinese adult skiers, providing a useful reference for ski safety management and training.

## Introduction

High-speed downhill sports (e.g., snowboarding, skiing, and mountain biking) take place on rugged terrain and uncertain weather conditions. They are considered high-risk activities potentially causing serious injuries and accidents (Thomson et al., 2014). The incidence of injuries in snow sports is extremely high, with a rate of 3.49 injuries in 1,000 sliders per day (Fu et al., 2022). There were 326 injuries reported at the 2022 Winter Olympics, with a damage rate of 11% (Gao et al., 2022). Data indicate higher injury levels in the last four Winter Olympics. Professional athletes have higher injury rates than non-professional (Yang et al., 2021). There are many influencing factors, e.g., a higher tendency to take risks and sensation seeking.

Zuckerman (2007) believed that sensation-seeking is a common characteristic among those who engage in dangerous sports, it includes the pursuit of novel and intense feelings or experiences produced by various stimuli, and their willingness to take risks for them. Motivations for participating in high-risk sports are thought to have individual differences, but thrill-seeking is a common motivation for almost every participant (Gomà I et al., 2012). High sensation-seeking individuals are believed to be prone to engaging in risky behaviors, e.g., DUI, reckless driving (Zuckerman and Neeb, 1980), smoking, alcohol abuse, drug abuse, and crime. They tend to prefer risky locations during their excursions (Kovačić et al., 2023). Low sensation-seeking individuals have a low probability of engaging in risky activities and behaviors (Gambatese et al., 2017).

Recent studies have expanded the understanding of sensation seeking in sports by incorporating cultural and contextual perspectives. For example, Chen et al. (2024) demonstrated that cultural values, such as collectivism, play a significant role in shaping sensation-seeking tendencies among Chinese adolescents. This finding suggests that risk-taking behavior may vary across different cultural settings. Kapetanovic et al. (2023) further highlighted the moderating role of temperament in the relationship between sensation seeking and subsequent substance use, emphasizing the importance of exploring sensation seeking within specific cultural and environmental contexts.

Measurement tools for sensation seeking include the UPPS (urgency, premeditation, perseverance, and sensation seeking) Impulsive Behavior Scale (Whiteside and Lynam, 2001) and the *Zuckerman-Kuhlman Personality Questionnaire* (*ZKPQ*; Zuckerman et al., 1993). They are designed to measure sensation-seeking across a range of life domains (social, occupational, physical, etc.). The measurement tools investigate sensation seeking in general situations and may not be applicable in high-risk ski sports situations. Slanger and Rudestam (1997) suggested that people who engage in high-risk snow sports may cluster too closely in the high scores of the *SSS* for their higher sensation seeking. General sensation-seeking scales lack the discrimination to differentiate consecutive individuals with high scores. Thomson et al. (2012) noted that ski participants’ motivations differ from excitement and adventure generated in a specific activity. They are more likely to be related to the level of sensation seeking within overall personality traits. Widely used sensation-seeking scales do not clearly express the specific risk and excitement associated with skiing and snowboarding. Therefore, Thomson developed the *CSSQ-S* related to risky behavior and published his psychometric results (Thomson et al., 2012). The *CSSQ-S* narrows the scope of questions to specific skiing and snowboarding. This scale has been widely used in studying sensation-seeking, injury, and risk-taking in skiing and snowboarding over the past decade (Marengo et al., 2017; Maher et al., 2015; Thomson and Carlson, 2015; Thomson et al., 2014). It has been translated into Italian (Claudia et al., 2018) and Turkish (Dinç and Demircan, 2019).

The risk is an important factor in previous studies. Sensation-seeking is highly related to risky behaviors, e.g., drug abuse, alcoholism, substance abuse, and sexual risks (Hu and Wang, 2019; Li, 2022). Risk perception is a cognitive activity that is modulated by situational and observer characteristics. It describes people’s subjective attitudes and intuitive judgments about objective risks (Huang and Wu, 2017; Hunter, 2002). Low sensation seekers reported higher levels of anxiety when faced with risks, while high sensation seekers reported positive arousal. This means that low sensation seekers perceive risk more strongly. A person may decide that the behavior (e.g., skiing at high speed) carries an acceptable risk. It depends on the perceived benefit of an activity (e.g., a rush of excitement). High sensation-seeking individuals may take greater risks to achieve optimal arousal levels. Risks include the possibility of negative outcomes. Injury is the most possible negative outcome, and low levels of risk perception are significantly associated with the increased risk of injury (Kontos, 2004). Given the complex relationship between sensation seeking, personality traits, and cultural background, more research is necessary to explore these interactions and their implications for injury prevention.

It is widely accepted that highly sensation-seeking individuals are more likely to be injured. Thomson et al. (2012) found that sensation seeking is significantly positively related to injury rates over three seasons with predictivity. An investigation into the National Collegiate Athletic Association (NCAA) found that student-athletes may make themselves more vulnerable to injury and conceal concussion symptoms to continue participating. This increases the risk and extent of injury (Graupensperger et al., 2018). Notably, the relationship between sensation seeking and injury is complex and influenced by other factors, e.g., personality traits, social environment, and cultural background. Further research could delve deeper into the mechanisms of the relationship and provide more targeted interventions for injury prevention.

Exploring sensation seeking in high-risk sports helps identify psychological processes that are particularly relevant to the risky behaviors of risky people. Risk-seeking athletes can be reminded of injury prevention in downhill sports by identifying them. However, there is a lack of effective tools to assess Chinese skiers and snowboarders’ sensation seeking in skiing and snowboarding. At present, most measurement tools for sensation seeking are developed from an overall perspective. When these tools are used on skiers, more errors may occur. Meanwhile, this study provides a more reliable measurement tool for Chinese researchers in the same field. Therefore, the work aimed to verify the reliability and validity of the CSSQ-S among Chinese adult skiers and snowboarders.

The work aimed to verify the reliability and validity of the *CSSQ-S* among Chinese adult skiers and snowboarders. The factor structure of this scale was expected to be similar to the original one, and its reliability was to be acceptable. EFA and CFA were used to explore its structure to achieve this goal. The relationship among the *CSSQ-S*, *Zuckerman-Kuhlman Personality Questionnaire-Impulsive-Sensation-Seeking (ZKPQ-ImpSS)*, *DOSPERT*, and *RPIQ* was examined to study the construct validity of the scale. It was assumed that the total score of the *CSSQ-S* was positively correlated with the *ImpSS* and negatively correlated with the *DOSPERT* and *RPIQ*.

## Methods

### Participants

The local ethics committee has approved this research protocol (2023207). Snowball sampling (Streeton et al., 2004) was used to collect data online and offline. We acknowledge this limitation and have carefully considered its potential impact on the generalizability of our findings. However, as Sherman (2024) argues, convenience sampling remains valid if the sample is unrelated to the theoretical issue, and we believe it does not compromise our conclusions. Initial samples distributed to college students were completed by friends, classmates, and acquaintances with experience in skiing and snowboarding. A total of 515 skiers and snowboarders aged 18–40 participated in the work (Wu, 2023). The inclusion criteria were as follows: (a) non-beginners (experiencing skiing or snowboarding proficiently many times), with at least one year of snowboarding or skiing experience; (b) age between 18 and 40. Sensation-seeking is believed to decrease after the age of 40 (Zuckerman and Neeb, 1980). Offline participants (*N* = 205) were collected from July 15 to July 18, 2023. Online participants (*N* = 310) were collected from July 20 to July 26, 2023.

**Figure 1:**
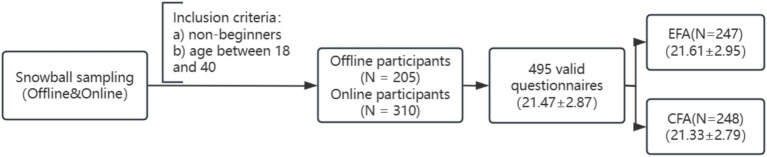
Participant flow diagram.

There were 495 questionnaires after filtering out invalid ones, with an effective recovery rate of 96.1%. The average age of participants was 21.471 ± 2.87. There were 24 first-level athletes, 90 s-level athletes, 132 third-level athletes, and the rest had no level. A total of 191 people were good at snowboarding, 180 people at skiing, and the rest were proficient in both among the total samples. Participants were divided into two sample groups for EFA and CFA, respectively (Figure 1).

Sample 1: 247 samples for EFA, with an average age of 21.61 ± 2.95. The subjects included 174 men and 73 women. Sample 2: 248 for CFA, with an average age of 21.33 ± 2.79. The subjects included 169 men and 79 women. (Table 1).

**Table 1:** Participant statistics.

Demographic variables	Sample 1 (n = 247)				Sample 2 (n = 248)			
	Min	Max	Mean	SD	Min	Max	Mean	SD
Age (year)	18	28	21.61	2.95	18	28	21.33	2.79
Gender^a^	1	2	1.30	0.46	1	2	1.32	0.47
Injury frequency over the past year	0	4	1.14	1.05	0	4	0.99	1.02
Years receiving training	1	13	4.43	2.67	1	10	4.57	2.51

^a1 = Male, a2 = Female; Mean is reported.^

### Sample size calculations

The sample size was determined based on statistical power analysis. Assuming a medium effect size (d = 0.5), a significance level ( *α* ) of 0.05, and a desired statistical power (1 − *β* ) of 0.80, the minimum required sample size for this study was calculated using G*Power software to be N = 176 for two groups. Given that our final sample consisted of 495 participants (with 247 and 248 for EFA and CFA, respectively), the sample size exceeded the minimum requirement, ensuring sufficient statistical power. Additionally, the sample was representative of Chinese skiers aged 18–40 with at least one year of skiing experience, making it appropriate for exploring sensation seeking and injury risk in this specific population.

## Materials

### Contextual Sensation-Seeking Questionnaire for Skiing and Snowboarding (CSSQ-S)

The original *CSSQ-S* consisted of 10 items and shared one factor (Thomson et al., 2012). Participants were asked to complete a 5-point Likert questionnaire ranging from 1 (strongly disagree) to 5 (strongly agree). Cronbach’s *α* of the original scale was 0.88.

### Zuckerman-Kuhlman Personality Questionnaire-Impulsive-Sensation-Seeking (ZKPQ-ImpSS)

The *ZKPQ* is a 99-item true-false scale. It is used to assess impulse-sensation-seeking, aggression/hostility, sociability, neuroticism/anxiety, and activity (Zuckerman et al., 1993). The I*mpSS Subscale* was used with 19 items. A two-point scoring system was used, with “no” scored as 0 point and “yes” scored as 1 point. *ImpSS* represented a lack of planning and a tendency to act impulsively without thinking. It also indicated a desire to seek out experiences or to take risks for excitement and novelty. Cronbach’s *α* of the *ImpSS Subscale* was 0.888.

### Domain-Specific Risk-Taking (DOSPERT) Scale -risk perception subscale

Weber et al. (2002) constructed a *Domain-specific Risk-taking Scale* to assess risky preferences and perceptions in social, entertainment, financial, health/safety, and ethical domains. The work adopted the revised version of Wu and Cheung (2014), which removed the assessment of the financial domain. There were 24 items with society, entertainment, health/safety, and morality. A 7-point Likert scale was used ranging from 1 (not at all risky) to 7 (extremely risky). It could be used to measure risk perceptions and the likelihood of engaging in risky activities or behaviors in four specified domains.

The *Risk Perception Subscale* of *the DOSPERT* was used. People’s risk perception rather than risk-taking was examined when the option was risk level (Not at all Risky to Extremely Risky)—the intuitive assessment of risk (Blais and Weber, 2006). Cronbach’s *α* of society, entertainment, health/safety, and morality were 0.836, 0.802, 0.758, and 0.827, respectively. Cronbach’s *α* of the main scale was 0.927.

### Risk, Pain, and Injury Questionnaire (RPIQ)

The *RPIQ* has 12 questions, consisting of tenacity, pressure tolerance, and rational choice (Liu et al., 2023). A *4-point Likert Scale* is used ranging from 1 (strongly agree) to 4 (strongly disagree). Tenacity refers to resilience in the face of risk, pain, and injury. Pressure tolerance refers to continuing to compete under pressure from coaches and spectators after an injury. Rational choice refers to an athlete’s willingness to accept the risks of sport. Scores reflect people’s attitudes toward risks, pain, and injuries they face in sports. Cronbach’s *α* of tenacity, pressure tolerance, and rational choice in the work were 0.685, 0.830, and 0.864, respectively. Cronbach’s *α* of the main scale was 0.872.

### Demographic questionnaire

Participants were asked to provide gender, age, education level, sport level, years of training, expertise in snowboarding or skiing, and the number of injuries sustained during sports in the past six months. *The Number of Injuries* reported injuries that skiing or snowboarding ability impaired for at least one day (a common definition in injury epidemiology; Goldberg et al., 2007; Nicholl et al., 1995).

### Procedure

The work obtained authorization for scale revision from Dr. Thomson. The original *CSSQ-S* was translated into Chinese following a rigorous translation and back-translation procedure to ensure cultural and linguistic appropriateness. Specifically, two English professors, Min Zhao and Xinxin Yu, and two sports psychology professors, G.B.D (One of the authors) and Ali Yang, independently translated and modified the original scale into Chinese. Subsequently, two bilingual professors of sports psychology, Ruosong Chang and Jinfei Ma, back-translated the Chinese version into English. The back-translated English version was compared with the original scale and revised to improve the accuracy and consistency of the translation. To ensure item accuracy, two sports injury experts, Yang Cheng and Feng Guo, reviewed the translated items. Finally, two psychology professors, Long Sun and Ye Tian, along with five psychology graduate students, assessed the content validity to adapt the scale for use in the Chinese context.

The item “I like to ski/ride out of bounds” was translated as “I like to slide out of the safe area” because the term “out of bounds” is not commonly understood by Chinese skiers. Instead, “safe area” is a more familiar concept that matches the terminology used in safety instructions at Chinese ski resorts; The original sentence “a cliff jump of more than 15 feet is not too high for me” was translated as “a cliff jump of more than 4 meters is not too high for me,” preserving the original meaning while adapting the measurement unit to a culturally appropriate standard. Thirty college students specializing in skiing were randomly selected to complete the scale to facilitate understanding by Chinese people. A final questionnaire was developed (inter-rater reliability of 0.71–0.89), containing 10 items. Participants completed informed consent forms and demographic information to complete the questionnaire. Online and offline were combined.

### Data analysis

Data were analyzed using SPSS26.0. Sample 1 was used to examine the statistical properties of the items, e.g., means, standard deviations, corrected item-total correlations, factor loadings, and commonalities. SPSS26.0 was used to conduct EFA on sample 1 to determine the factor structure of the *CSSQ-S*. Reliability analysis was performed to check the internal consistency of *RPIQ* factors, and Cronbach’s *α* was greater than expected 0.6. CFA was conducted using sample 2 via Amos 23.0 to assess the model suitability of the *CSSQ-S*.

The overall sample was used to analyze the Pearson correlation between the dimensions of the *CSSQ-S*, *ImpSS*, and *DOSPERT* and those of the *RPIQ* to test the construct validity. SPSS26.0 was used to conduct regression analysis on the overall sample to explore other possible relationships among variables. The overall sample was used to conduct a mediation effect analysis through PROCESS3.5 to explore the relationship among the *CSSQ-S*, *DOSPERT*, and sports injuries.

### Effect size calculations

To complement *p*-values, effect sizes were calculated for all statistical analyses. For correlation analyses, *r^2^* values were reported to indicate the proportion of shared variance. For regression analyses, *R^2^* was used to represent the total variance explained by the predictors, while Cohen’s *f ^2^* was used to measure the effect size of individual predictors. For mediation analyses, standardized path coefficients (*β*) and the proportion of mediated effect were reported.

## Results

### Descriptive statistics

Sample 1 is used to analyze the descriptive attributes of the items. Table 2 shows mean, standard deviation, corrected item-total correlation, factor loadings, and commonalities. Mean for each item ranges from 2.67 to 3.0, indicating no ceiling or floor effects. The total correlation coefficient of each item is greater than 0.3 after correction.

**Table 2:** Descriptive statistics of CSSQ-S items.

Items	Mean	SD	Corrected item-total	Factor loading	Community
1	2.94	1.28	0.70**	0.76	0.58
2	3.00	1.30	0.67**	0.74	0.55
3	2.81	1.24	0.67**	0.74	0.54
4	2.68	1.22	0.69**	0.76	0.58
5	2.80	1.24	0.67**	0.74	0.55
6	2.99	1.27	0.75**	0.81	0.65
7	2,79	1.21	0.62**	0.70	0.48
8	2.80	1.25	0.66**	0.73	0.53
9	2.93	1.25	0.71**	0.78	0.61
10	2.67	1.25	0.65**	0.72	0.52

*Indicates *p* < 0.05; ** indicates *p* < 0.01.

### Exploratory factor analysis

EFA was conducted on the *CSSQ-S* using sample 1. *KMO =* 0.913, *X^2^* = 1303.23, *df* = 45, and *p* < 0.001, indicating that the data was suitable for EFA. One factor with an eigenvalue greater than 1 was extracted through principal component analysis and scree plots. Its eigenvalue was 5.59, and the variance explanation rate was 55.91%. The factor loadings of each item in the component matrix ranged from 0.70 to 0.81 (Table 2).

### Confirmatory factor analysis

The model fitting of the *CSSQ-S* was evaluated by Amos 23.0 using verifiable CFA from sample 2. The model fit of the original 10-item questionnaire was checked. The model fit index reached the ideal range (*CMIN* = 87.70, *DF* = 35, *CMIN/DF* = 2.51, *CFI* = 0.96, *TLI* = 0.95, and *RMSEA* = 0.078). According to the criteria proposed by Hu and Bentler (1999), *CFI* and *TLI* > 0.9 and *RMSEA* < 0.08, indicating that the model-fitting index of the *CSSQ-S* was acceptable.

### Reliability

Sample 1 was used to test the reliability of the *CSSQ-S*. The internal consistency (Cronbach’s *α*) of the scale was 0.91. The split-half reliability (Spearman-Brown) of the scale was 0.89, indicating that the reliability of the scale was acceptable.

### Criterion-related validity

The *DOSPERT* and *RPIQ* are selected as the criterion questionnaires, and the criterion-related validity test is conducted on the overall sample. The CSSQ-S was significantly positively correlated with ImpSS (*r* = 0.580, *p* < 0.01), indicating that 33.6% (*r^2^* = 0.336) of the variance in sensation seeking is shared between these measures. It was also negatively correlated with DOSPERT (*r* = −0.577, *p* < 0.01, *r^2^* = 0.333) and RPIQ (*r* = −0.658, *p* < 0.01, *r^2^* = 0.433) (Table 3).

**Table 3:** Correlations among the *CSSQ-S*, *ImpSS*, *DOSPERT*, and *RPIQ.*

	1	2	3	4	5	6	7	8	9	10	11
1. *CSSQ-S*	1										
2. *ImpSS*	0.580	1									
3. *DOSPERT*	0.577	0.500	1								
4. Society	0.473	0.375	0.820	1							
5. Recreation	0.544	0.455	0.901	0.691	1						
6.Health/Safety	0.488	0.441	0.907	0.678	0.754	1					
7. Ethics	0.474	0.443	0.800	0.416	0.650	0.687	1				
8. *RPIQ*	0.658	0.506	0.561	0.566	0.497	0.474	0.376	1			
9. Tough	0.446	0.345	0.356	0.329	0.332	0.292	0.265	0.777	1		
10. Pressure	0.452	0.354	0.395	0.345	0.357	0.331	0.317	0.814	0.662	1	
11. Choice	0.655	0.499	0.564	0.617	0.487	0.483	0.334	0.868	0.457	0.484	1

The *p*-values for all the data are less than 0.01.

### Analysis of the mediating effect between the *CSSQ-S* and sports injuries

#### Regression analysis

A linear regression analysis was conducted on the overall sample, using sensation seeking and risk perception as independent variables. The dependent variables were the number of injuries in the past six months and risk perception, respectively.

Before conducting the regression analysis, we checked for potential multicollinearity among predictor variables using Variance Inflation Factor (VIF) values. All VIF values were below 5, indicating no significant multicollinearity. Additionally, the condition index was below 30, further confirming that multicollinearity was not a concern. We tested the key assumptions of regression analysis. The Shapiro–Wilk test confirmed that residuals followed a normal distribution (*p* > 0.05), the Breusch-Pagan test indicated no heteroscedasticity (*p* > 0.05), and the Durbin-Watson statistic was close to 2, suggesting no significant autocorrelation. These results verified that all regression assumptions were met. The independent variables explain 14.2, 10.5, and 33.2% of the dependent variables. It shows that all three independent variables can predict the corresponding dependent variables to a certain extent (Table 4).

**Table 4:** Results of regression analysis.

Independent variables	Dependent variables	β	t	*p*	Adjusted R^2^	F
Risk perception	Sensation seeking	−0.577	−15.704	<0.001	0.332	*F* = 246.63 *p* < 0.001
Injury frequency	Sensation seeking	0.380	9.113	<0.001	0.142	*F* = 83.04 *p* < 0.001
Injury frequency	Sensation seeking	0.287	5.678	<0.001	0.158	*F* = 47.31 *p* < 0.001
	Risk perception	−0.160	−3.169	0.002		

The model explained 15.8% (R^2^ = 0.158) of the variance in injury frequency. Cohen’s f^2^ was calculated to assess the effect size of sensation seeking and risk perception on injury frequency:


$$f^{2}=\frac{R_{\mathrm{inclusion}}^{2}-R_{\mathrm{exclusion}}^{2}}{1-R_{\mathrm{inclusion}}^{2}}=0.187$$

This indicates a medium effect size according to Cohen’s guidelines (*f^2^* = 0.02, small; *f^2^* = 0.15, medium; *f^2^* = 0.35, large).

#### Analysis of the mediating effect

PROCESS 3.5 is used for model 4 to explore the mediating effect of risk perception. The mediating model was selected based on the theoretical framework and prior research, which highlights risk perception as a key mediator between sensation seeking and injury outcomes in high-risk sports. The total score of contextual sensation seeking in skiing and snowboarding is the independent variable; the total score of risk perception of the *DOSPERT* is the mediating variable; the number of injuries in sports in the past six months is the dependent variable.

**Figure 2:**
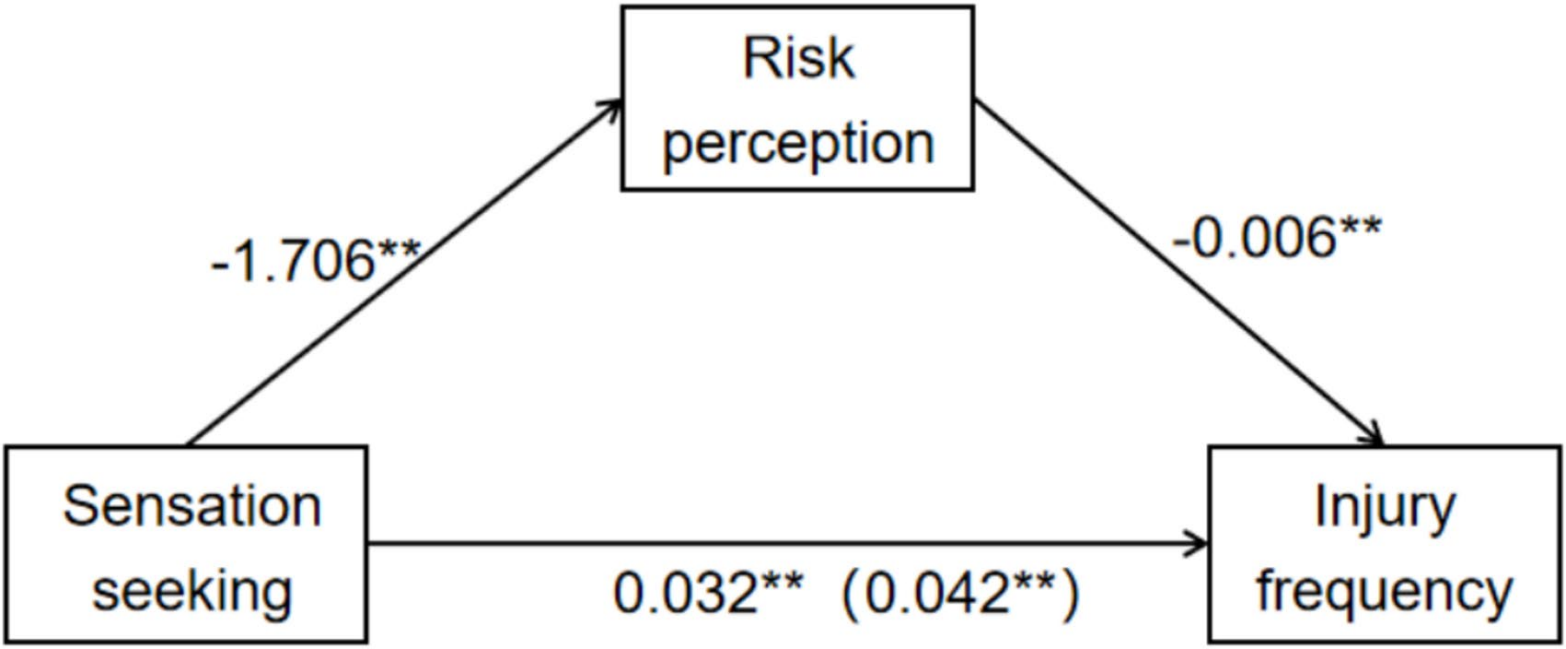
The mediating role of risk perception: how sensation seeking influences the number of skiing – related injuries.

Sensation seeking significantly positively predicts the number of injuries and negatively predicts risk perception. Sensation seeking has a significant negative predictive effect on the number of injuries (*β* = 0.032, *p* < 0.01) after adding the mediating variable of risk perception (Figure 1). Sensation seeking significantly predicted the number of injuries (*β* = 0.042,*p* < 0.01), with a direct effect of *β* = 0.032 and an indirect effect (via risk perception) of *β* = 0.010. Risk perception mediated the relationship, accounting for 24.34% of the total effect (Table 5). The standardized path coefficients indicate a small but significant mediation effect. The mediating effect value is 0.010, and the 95% confidence interval is [0.004, 0.017] (Figure 2).

**Table 5:** Bootstrap test and amount of the mediating effect (the number of injuries in sports in the past six months as the dependent variable).

Pathways	Effect	Boot SE	Boot LLCI	Boot ULCI	Proportion
Total	0.042	0.005	0.033	0.051	100%
Direct	0.032	0.005	0.022	0.042	75.66%
Mediating	0.010	0.003	0.004	0.017	24.34%

Bootstrapped confidence intervals are used to assess the stability and reliability of the mediating effect.

## Discussion

The work applies the *CSSQ-S* to Chinese skiers and snowboarders to evaluate whether it accurately assesses their sensation-seeking in snowboarding and skiing. Results indicate that the reliability and validity of the Chinese *CSSQ-S* are satisfactory. Each fit index meets psychometric standards, indicating that the structure of the scale is clear. The *CSSQ-S* is not redundantly related to the established scale according to the correlation analysis. Chinese skiers or snowboarders may lack foresight and planning when skiing (*ImpSS* impulsiveness). They desire new adventures and exciting experiences (*ImpSS* sensation seeking). A survey study conducted by Weishaar et al. (2023) among extreme sports enthusiasts, including skiers, found that lack of foresight is a significant independent predictor of injuries after controlling for other variables. This finding is consistent with the results of the present study. Similarly, Frühauf et al. (2017) suggested that experienced extreme sports participants do not deliberately seek risks but instead minimize risks by relying on their knowledge and skills. An interesting point found in this study is that injury frequency was positively correlated with sports experience, which aligns with previous research. This correlation may be attributed to physical function decline and cumulative sports-related injuries in experienced skiers (Ling et al., 2022). There is a significant negative correlation (*r* = −0.58 and *p* < 0.001) between the *CSSQ-S* and *DOSPERT*. Chinese skiers’ scores of the *CSSQ-S* are moderately negatively correlated with risk attitude scores in society, recreation, health/safety, and moral domains. High sensation-seeking skiers have a higher target level of acceptable risk (Zuckerman, 2007) and are more likely to identify risky behaviors as lower risk. The *CSSQ-S* has a significant negative correlation with the three factors of the *RPIQ*—resilience, pressure tolerance, and rational choice. The *CSSQ-S* is highly negatively correlated with rational choice (0.6 ≤ |*r*| <0.8). High-risk sports individuals are willing to take risks associated with a sport for their higher sensation seeking. They are more resilient in the face of risk, pain, and injury, and more willing to compete under pressure from coaches and spectators after an injury.

Low sensation seekers exhibit more cautious behaviors during exercise, which reduces the risk of injury. Our study found that risk perception can negatively predict the number of injuries. The lower the risk perception score, the higher the number of injuries experienced by skiers. Skiers with lower risk perception are more likely to experience accidents while skiing because they fail to fully appreciate potential hazards. Risk perception is associated with substance-use disorders, injuries, and risky behaviors, i.e., overeating, DUI, riding with drunk drivers, and low seatbelt use. These results highlight the critical role of risk perception in determining the number of injuries and other risky behaviors among skiers. An individual’s level of perception of potential danger may influence their behavioral decisions in risk-taking and dangerous situations, which directly affect their safety and health status. The work explores the mediating role of risk perception between sensation seeking and the number of injuries among Chinese skiers and snowboarders. Sensation-seeking affects the number of injuries experienced by skiers by affecting the level of risk perception. Sensation-seeking affects injury risk through direct behaviors as well as behavioral decisions by affecting an individual’s perception of risk. Sensation-seeking scores are higher among those of higher risks (Ruedl et al., 2012). Higher-risk individuals tend to seek sensory novelty and excitement. This sensation-seeking behavior makes them more likely to ignore potential risks and therefore score lower on risk perception. Cautious individuals are more sensitive to potential risks and therefore score lower on sensation seeking. Skiers who are high in sensation-seeking underestimate potential risks because they are more focused on pursuing thrills and pleasure. Research shows there are greater risks associated with these sports. However, participants believe that they can face various dangerous situations. High self-efficacy makes them overconfident in their ability to face dangers and reduce their vigilance. Low sensation-seeking skiers focus on safety and control. They are more sensitive to potential risks and more inclined to avoid potentially dangerous situations. This makes them more likely to be cautious while skiing, which reduces the risk of injury.

The study also found that the CSSQ-C showed a high correlation with the recreational dimension of DOSPERT, while its correlation with other dimensions was relatively low. A reasonable explanation for this could be that skiing itself is an extreme sport, and the CSSQ-C is specifically designed to measure sensation-seeking behavior in skiing contexts. Similarly, the recreational dimension of DOSPERT includes items related to extreme sports, which likely resulted in a high correlation. Meanwhile, factors such as the participants’ cultural background and education level were consistent. Moreover, it is widely acknowledged in academic research that Chinese individuals tend to be more conservative (Beattie et al., 2022; Chen, 2022), which may explain the lower and more uniform correlations in other dimensions.

The mediating effect makes sensation seeking a direct factor and affects an individual’s risk of injury at a deeper level by changing their perception of risk. It has important implications for the safety management of skiing and skiers’ behavioral decisions. Skiers can be reminded to be fully aware of the potential risks when participating in the sport, especially for individuals who tend to seek thrills and adventures. The findings serve as a guide to help develop more targeted measures to reduce skiers’ risk of injury in terms of ski safety management and training. Based on the findings, we propose practical safety measures: For instructors and coaches, use the CSSQ-S to assess students’ sensation-seeking levels. Focus on safety education and personalized training for high-scorers, incorporating risk perception training through accident case studies and simulated scenarios. For policymakers, enforce stricter ski-resort safety regulations, including slope-specific rules, improved protective facilities, and regular inspections. Promote safety awareness through resort posters, online content, and safety workshops for skiers.

### Limitations and future research

Several limitations should be acknowledged in this study. First, the sample size was relatively small, which may affect the generalizability of the findings. Future research should expand the sample to include a more diverse population of skiers. Second, the age range of participants was limited to adults, excluding younger skiers. Future studies could include underage participants to verify the applicability of the CSSQ-S across different age groups in China. Third, part of the data was collected through offline self-reports, which may introduce self-report bias, such as social desirability effects, potentially leading to discrepancies between reported and actual behaviors. Future studies should consider using objective data sources (e.g., injury records) to minimize this bias. Finally, the study relied on retrospective self-reported injury data from the past six months, which may be subject to recall bias and inaccuracies. A prospective longitudinal design that tracks participants’ future injuries would provide stronger evidence for the relationship between sensation seeking and injury risk in high-risk sports.

The findings have practical implications. The work takes the first step in applying the *CSSQ-S* to China. The measurement of sensation-seeking focuses on high-risk sports. Chinese adult skiers and snowboarders are the subjects. It is recommended that professional athletes and ski enthusiasts be distinguished in the future to test their reliability and validity, respectively. Snowboarding and skiing are similar to other high-speed downhill sports. The items of the scale can be adapted to measure sensation seeking in other high-risk sports, e.g., downhill mountain biking and kayaking. Narrow slides and obstacles in these sports affect the excitement and risk of the activities. The relationship among sensation seeking, risk perception, and injury is explored. Increasing the risk perception to reduce injury is meaningful for skiers and snowboarders with high sensation seeking.

## Conclusion

The Chinese *CSSQ-S* has sufficient psychometric properties and can be used as a reliable and valid tool to measure the sensation-seeking level of Chinese adult skiers and snowboarders. The work reveals significant correlations among the *CSSQ-S*, *ImpSS*, *DOSPERT*, and *RPIQ* as well as the relationship between the *CSSQ-S* and skier injuries. Moreover, it has the potential to be adapted for other high-risk sports, such as downhill mountain biking, rock climbing, and white-water kayaking, aiding professionals in developing targeted safety strategies. Additionally, the CSSQ-S could be valuable in longitudinal studies, allowing researchers to track changes in sensation-seeking tendencies and risk-taking behaviors over time. This would provide deeper insights into their long-term impact on injury risk and help create more effective injury-prevention programs.

## Data Availability

The original contributions presented in the study are included in the article/Supplementary material, further inquiries can be directed to the corresponding author.
