# Supplementary material for: Validation of the Contextual Sensation-Seeking Questionnaire for skiing and snowboarding among Chinese adult skiers and its relationship with risk-taking behavior
Source: Front Psychol. 2025 Feb 24;16:1410930. doi: 10.3389/fpsyg.2025.1410930 (PMC11891213; doi:10.3389/fpsyg.2025.1410930)
Supplement: Supplementary file 1 [file Data_Sheet_1.docx]

**Revised Version of CSSQ-S**

|  | 非常不同意 | 不同意 | 一般 | 同意 | 非常同意 |
| --- | --- | --- | --- | --- | --- |
| 1.我喜欢快速滑雪。 |  |  |  |  |  |
| 2.我喜欢挑战以前从未滑过的雪道。 |  |  |  |  |  |
| 3.即使看不到前方的情况(例如有很高的悬崖)，我也喜欢开始滑行。 |  |  |  |  |  |
| 4.我喜欢滑出安全区域。 |  |  |  |  |  |
| 5.即使不确定落点的情况，我也喜欢尝试跳跃。 |  |  |  |  |  |
| 6.我喜欢在滑雪时挑战自己的极限。 |  |  |  |  |  |
| 7.如果我失去控制，我不会试图立刻减速，而是顺其自然。 |  |  |  |  |  |
| 8.如果唯一的下山路线是通过一个狭窄的笔直通道下山，哪怕我知道速度会很快，我也会毫不犹豫地冲下去。 |  |  |  |  |  |
| 9.我总是试图找到新的、令人兴奋的下山方式。 |  |  |  |  |  |
| 1. 4米多的悬崖跳跃对我来说不算太高。 |  |  |  |  |  |
